# Effect and safety of intraoperative intraperitoneal chemotherapy on patients suffering from colorectal cancer

**DOI:** 10.1186/s12957-021-02197-3

**Published:** 2021-03-22

**Authors:** An Shang, Shuang Wang, Yongping Yang, Liping Li, Zeyun Zhao, Donglin Li, Yu Guo, Min Wang

**Affiliations:** 1 Colorectal Section, Department of Surgery, The Second Hospital of Jilin University, No. 218, Ziqiang Dist, Changchun, 130041 Jilin China; 2 Department of Dermatology, The Second Hospital of Jilin University, No. 218, Ziqiang Dist, Changchun, 130041 Jilin China; 3 Department of Hematology and Oncology, The Second Hospital of Jilin University, No. 218, Ziqiang Dist, Changchun, 130041 Jilin China

## Abstract

**Background:**

Colorectal cancer (CRC), the third most commonly diagnosed malignant carcinoma and the third most common cause of carcinoma-related mortality, continues to be a major international health problem. And approximately 33% of patients suffer from recurrence after radical surgery. Free malignant cell implanting in the peritoneum is generally accepted as one of the main reasons of such outcome. We did this present clinical study with the aim of evaluating the effects and safety of intraoperative intraperitoneal chemotherapy (IOC) on patients suffering from colorectal cancer, with hoping to find a novel, effective, and available approach to deal with malignant cell implanting during surgeries.

**Methods:**

In total, 391 patients who went through colorectal radical surgery were considered eligible between June 1, 2017, and December 31, 2018. 220 patients were treated with surgery without IOC, while other 171 patients received surgery plus IOC. Clinical characteristics, operative findings, postoperative short-term outcomes, disease-free survival (DFS), and overall survival (OS) were compared between these above 2 groups in the selected population.

**Result:**

The present research included 391 patients (251 men and 140 women) who underwent surgery without IOC (*n* = 171) or surgery plus IOC (*n* = 220), with a mean (SD) age of 60.4 (9.7) years in the surgery without IOC group and 60.6 (8.7) in the surgery plus IOC group (*P*=.85). No significant differences were witnessed between the two groups in surgery-related information and postoperative complications. It is worth noting that IOC independent of other factors was associated with a favor prognosis in CRC patients with stage II/III (HR 0.50, 95%CI 0.30–0.82, *P*=.006). Moreover, for patients with stage II colorectal carcinoma, DFS did not differ between two groups (*P*=.553, Kaplan-Meier log-rank), and OS was no exception. In stage III CRC patients, the estimated DFS rate for patients receiving IOC was 82.2% and patients without IOC was 66.4% after 3 years, which demonstrated that IOC was associated with a favorable prognosis in stage III patients (*P*=.012, Kaplan-Meier log-rank). Furthermore, the differences were still remained between the two groups when considering the influence about postoperative chemotherapy (*P*=.014, Kaplan-Meier log-rank). IOC can also significantly improve patients’ overall survival whether they get treatment with POC (*P*=.006, Kaplan-Meier log-rank; *P*=.025, Kaplan-Meier log-rank).

**Conclusions:**

In the present study, we have found that surgery plus IOC generated a favorable prognosis for stage III CRC patients but not stage II without any side-effects when the dosage of lobaplatin was 0.1g/L. As a new, safe, and simple procedure, IOC therapy is easily performed—and does not require any special devices or techniques. Thus, IOC is a promising and exciting therapeutic strategy for patients with CRC.

## Introduction

Following lung cancer and breast cancer in females and lung cancer and prostate cancer in males respectively, colorectal cancer, the third most commonly diagnosed malignant carcinoma and the third most common cause of carcinoma-related mortality, a major international health problem [1]. Although the total quantity of CRC is still the highest in Western countries, the incidence and mortality there tend to be stabilized or even decreased; however, the trend of morbidity and mortality seems to bet the opposite outcomes in many developing countries, such as China [2]. Currently, radical resection remains the reference-standard treatment for early and even advanced cancer. The standard strategy of treatment for CRC, besides radical resection, is intravenous chemotherapy. Nevertheless, approximately 33% of patients suffer from recurrence after radical surgery [3]. Extraordinary, peritoneal carcinomatosis, as a common type of CRC metastasis, has long been regarded as associating with poor prognosis for patients after radical surgery, and whose overall survival is even as poor as the multiple-organ metastases [4–6]. That outcome would partially due to the insensitivity to systemic chemotherapy for peritoneal metastasis [7].

Peritoneal-free cancer cell (PFCC) implanting in the peritoneum is generally accepted to be one of the main reasons of carcinoma recurrence [8]. Consequently, it is necessary to kill free malignancy cells before fixation on the peritoneum [9]. Cytoreductive surgery (CRS) with hyperthermic intraperitoneal chemotherapy (HIPEC) has been recommended as an alternative approach for patients who have undergone R0 resection and resist cancer recurrence in the peritoneum [10]. However, it cannot be ignored that high morbidity and mortality restrict the application of HIPEC [11–13]. It is worth noting that opening lymphatic channels during operation might spread viable cancer cells into the abdominal cavity, which certainly provides evidence of the effectiveness of intraoperative intraperitoneal chemotherapy [8]. However, the most suitable medicine for IOC is still controversial. Lobaplatin (chemical formula: C9H18N2O3Pt) exerts stronger anti-neoplastic effects with fewer adverse effects as a third-generation platinum anti-neoplastic agent [14]. And GSDME-dependent pyroptosis as a possible mechanism for lobaplatin to eradicate colorectal neoplastic cells has been confirmed [15]. Furthermore, several studies has indicated that perfusion chemotherapy with lobaplatin can suppress proliferation and peritoneal metastasis of colorectal cancer and promote a favorable prognosis for CRC without any side effects [16, 17]. Therefore, lobaplatin may be a relatively better choice for IOC.

Though intraoperative intraperitoneal chemotherapy as a new strategy to improve prognosis after R0 resection for CRC has been confirmed valid [18], further research is still needed to figure out whether it can prolong overall survival (OS) time, prevent peritoneal metastasis following the radical operation and any associated complications. We did the study with the aim of evaluating the effect and safety of intraoperative intraperitoneal chemotherapy on patients with colorectal cancer.

## Method

### Patients

The retrospective study has met with approval by the ethics committee of The Second Affiliated Hospital of Jilin University and performed in accordance with the Helsinki Declaration of World Medical Association, and the demand of patient informed consent was deserted because of the retrospective nature of this study. After rigorous screening, eventually, 391 patients who went through colorectal radical surgery were considered to be eligible from June 1, 2017, to December 31, 2018, in our department. The baseline characteristics of the selected patients, consisting of age, gender, diabetic mellitus, hypertension, tumor location, tumor size, pathologic T category, pathologic N category, TNM stage (based on the postoperative pathology), degree of differentiation, tumor pathologic type, postoperative adjust chemotherapy, and carcinoma embryonic antigen (CEA) before the operation was carefully collected from medical records. Primary locations of tumors were defined as the right colon (from the cecum to the transverse colon), left colon (from the splenic flexure to the rectosigmoid flexure), and rectum (15 cm from the anal verge).

The intra- and post-operation date, consisting of operation method, operation time, amount of intraoperative blood loss, time to first flatus, LOS (length of stay), abdominal pain, and laboratory results (such as white blood cell count, neutrophil count, neutrophil-lymphocyte ratio, hemoglobin, albumin, and albumin globulin ratio), 48 h after the operation was also collected through consulting patients’ medical notes. Follow-up information was obtained at an outpatient clinic of our center or using a telephone questionnaire directly. The final follow-up date for all of the cases was on December 1, 2020; Disease-free survival (DFS) is defined as the time from radical operation to recurrence of tumor or death, and overall survival (OS) is defined as the time from radical operation to death.

Visual Analogue Scale/Score (VAS), ranging from 0 to 10 (0, no pain; 1 to 3, mild pain [sustainable, sleep is not affected], 4 to 6, moderate pain [sleep is affected and painkillers are usually needed], 7 to 10, severe pain [Sleep is severely disrupted and painkillers are necessary]), was applied to evaluate the pain degree of postoperative patients [19]. In this study, the pain was defined as greater than 3 on the scale, considered to potentially affect emotional or physical functioning [20]. Postoperative complications (such as anastomosis or intra-abdominal bleeding, anastomosis leakage, abdominal cavity abscess, wound problems, intestinal obstruction, lymphatic leakage, cardiac disease, deep vein thrombosis, and pulmonary disease) were assessed through clinical manifestations, laboratory examination results, ultrasonography reports, and imaging findings. Furthermore, massive hemorrhage was defined as an amount of at least 300 ml. Patients with albumin levels below 30g/L were defined as hypoproteinemia.

### Inclusion criteria

The inclusion criteria were showed as follows: (1) age between 18 and 75 years; (2) pathologically diagnosed as colorectal carcinoma; (3) TNM stages II–III, diagnosed through postoperative pathology; and (4) patients underwent colorectal R0 resection.

### Exclusion criteria

The following exclusion criteria applied to patients in this research are (1) previous history of other systemic malignancies; (2) patients of familial adenomatous polyposis or human nonpolyposis CRC; (3) severe respiratory tract, liver, kidney, or cardiovascular disease; (4) the patients going through emergency surgery; and (5) the patients whose information cannot be collected accurately.

### Surgical procedure

Bowel preparation was performed by taking sulfate-free polyethylene glycol electrolyte powder orally 1 day before surgery. A standardized R0 surgical resection of colorectal carcinoma was then performed, and all surgical procedures were conducted with strict adherence to the National Ministry of Colorectal Cancer diagnosis and treatment standards. Different procedures were selected according to the location of carcinomas. Laparotomy or laparoscopy was chosen according to intraoperative findings. Peritoneal lavage, as an important procedure was normally performed using 1000 ml 0.9% saline solution after intestinal anastomosis, which was then absorbed completely. Finally, the excised specimen was sent to professional pathologists to identify the TNM stage.

### IOC

Lobaplatin was used for patients who underwent intraoperative intraperitoneal chemotherapy. Fifty milligrams of lobaplatin was dissolved in 500 ml 0.9% saline solution (SS) at a concentration of 0.1 g/L. The solution was then injected into an abdominal cavity through the drainage tube after the abdominal incision or laparoscopic port was closed. Vibrating abdomen adequately was routinely performed to make mixed solution distributing in the abdominal cavity evenly as far as possible. Finally, the mixed solution was discharged from abdominal cavity 5 h later. In the meanwhile, the drainage tube is closed to prevent the efflux of abdominal chemotherapy drugs. And whether patients received IOC is up to themselves or their family members before operative.

### Statistical analysis

The study was designed to evaluate the superiority in terms of disease-free survival (DFS) of combining radical surgery and IOC compared with surgery without IOC. Survival curves were created using the Kaplan-Meier method, and the differences between the two groups were compared using *t* texts and χ^2^ tests. Multivariate analyses were evaluated with Cox proportional hazards models. All *P* values calculated in the analysis were 2-sided, and *P* values less than 0.05 were considered statistically significant. Statistical analyses were performed using SPSS software, version 26.0 (IBM Corporation).

## Results

**Fig. 1:**
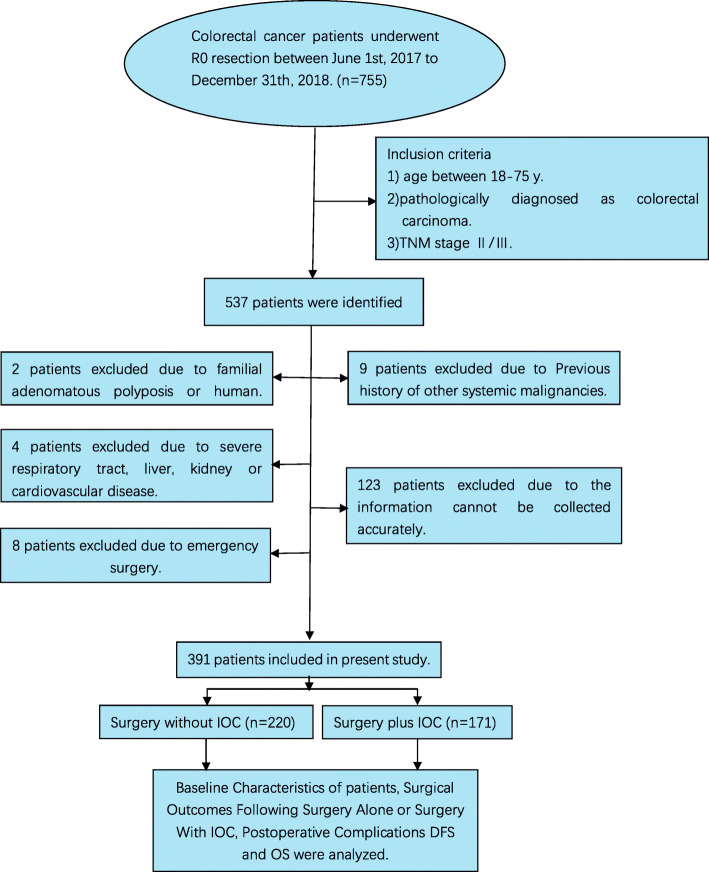
CONSORT diagram of patient flow

From June 1, 2017, to December 31, 2018, a total of 755 patients who underwent radical surgery for colorectal carcinoma in The Second Affiliated Hospital of Jilin University were collected, of which 537 cases were selected to further assessed ulteriorly according to the inclusion criteria. And 146 cases were excluded according to exclusion criteria (including 9 patients with previous history of other systemic malignancies; 2 patients with familial adenomatous polyposis; 8 patients undergoing emergency surgery; 4 patients with serious respiratory tract, liver, kidney, or cardiovascular disease; and 123 patients’ medical records unavailable) (Fig. 1). 391 cases were eventually enrolled in the present study eventually, with 220 patients assigned contents for the surgery without IOC group, and 171 assigned to the surgery plus IOC group.

The present research included 391 patients (251 men and 140 women) who underwent surgery without IOC (*n* = 171) or surgery plus IOC (*n* = 220), with a mean (SD) age of 60.4 (9.7) years in the surgery without IOC group and 60.6 (8.7) in the surgery plus IOC group (*P*=.85). Table 1 demonstrated that there was no statistical difference in the baseline clinical characteristics of the 391 patients between the two groups.

**Table 1:** Baseline characteristics of patients

Characteristic	Patients, no. Surgery without IOC (***N***=220)	Surgery plus IOC (***N***= 171)	***P*** value
Age, mean (SD), y	60.4(9.7)	60.6(8.7)	.85
Sex			
Male	137	114	.37
Female	83	57	
Hypertension			
Yes	57	49	.55
No	163	122	
Diabetic mellitus			
Yes	38	30	.94
No	182	141	
CEA, mean (SD), ng/mL	8.41(14.90)	9.07(15.41)	.67
Tumor location			
Right colon	64	39	.37
Left colon	48	40	
Rectal	108	92	
Tumor size, mean (SD), cm	4.8(1.6)	4.6(1.7)	.42
Pathologic T category			
T1	4	3	.84
T2	8	9	
T3	195	147	
T4	13	12	
Pathologic N category			
N0	96	80	.76
N1	93	66	
N2	31	25	
TNM stage			
II	96	80	.54
III	124	91	
Differentiation			
Well	6	4	.44
Moderate	204	154	
Poor	10	13	
Pathological type			
Tubular adenocarcinoma	209	159	.12
Mucinous adenocarcinoma	7	3	
Mixed adenocarcinoma	4	9	
Vascular invasion			
Yes	86	65	.83
No	134	106	
Postoperative chemotherapy			
Yes	122	92	.75
No	98	79	

Abbreviation: *IOC* Intraoperative intraperitoneal chemotherapy

Surgery-related information is presented in Table 2. Laparoscopy surgeries were performed in a large proportion of patients (79.0%), 172 laparoscopy operations and 48 open surgeries were performed in the surgery without IOC group, and 137 laparoscopy operations and 34 open surgeries were performed in combining surgery and IOC group. No significant differences were observed between groups in operation methods, ASA stage, operation time, and amount of intraoperation bleeding. Compared with the group of 171 patients receiving surgery without IOC, the group of 220 patients undergoing surgery plus IOC showed a similar trend in terms of time to first flatus (72.6[10.4] vs 72.8[9.7]; difference, −0.2; 95%CI, −2.2–1.8; *P*=.82), LOS (19.2[6.4] vs 18.6[5.3]; difference, 0.6; 95%CI, −0.6–1.8; *P*=.32), and postoperative laboratory results.

**Table 2:** Surgical outcomes following surgery without IOC or surgery with IOC

Outcome	Mean (SD) values surgery without IOC (***N***=220)	Surgery plus IOC (***N***=171)	Between-group difference (95% CI)	***P*** value
Operation method, no. (%)				
Laparoscopy	172(78.1)	137(80.1)		.64
Open surgery	48(21.9)	34(19.9)		
ASA				
II	123(55.9)	98(57.3)		.92
III	95(43.2)	71(41.5)		
IV	2(0.9)	2(1.2)		
Operation time, min	212.6(54.2)	211.6(46.2)	0.96(−9.30 to 11.16)	.85
Amount of intraoperation bleeding, ml	113(58)	114(47)	−0.3(−11.0 to 10.5)	.96
Time to first flatus, hour	72.6(10.4)	72.8(9.7)	−0.2(−2.2 to 1.8)	.82
LOS, day	19.2(6.4)	18.6(5.3)	0.6(−0.6 to 1.8)	.32
Postoperative laboratory results				
White blood cell count, ×109/L	10.68(2.67)	10.39(2.53)	0.30(−0.23 to 0.81)	.28
Neutrophil count, ×109/L	8.81(2.76)	8.57(2.52)	0.25(−0.28 to 0.78)	.36
Neutrophil ratio	0.819(0.073)	0.817(0.079)	0.002(−0.014 to 0.017)	.83
Hemoglobin, g/L	119(17)	119(21)	0.6(−3.2 to 4.4)	.75
Albumin, g/L	33.5(3.2)	33.7(3.1)	−0.2(−0.8 to 0.5)	.58
Albumin globulin ratio	1.36(0.18)	1.38(0.21)	−0.03(−0.06 to 0.01)	.20

Abbreviation: *IOC* Intraoperative intraperitoneal chemotherapy, *ASA* American Society of Anesthesiologists, *LOS* Long of stay

No perioperative deaths occurred both in the surgery without IOC group and the surgery plus IOC group (Table 3). No difference in abdominal pain was witnessed between the surgery without IOC group (84 of 220 patients [38.2%]) and surgery combined IOC group (74 of 171 patients [43.3%]) (difference, −5.1%; *P*=.31), nor in hypoproteinemia, anastomosis or intra-abdominal bleeding, anastomosis leakage, abdominal cavity abscess, wound problems, intestinal obstruction, cardiac disease, deep vein thrombosis, or pulmonary disease. Clavien-Dindo classification [21] was used to assess the severity of postoperative complications. There was no significant difference between two groups in I/II stage complications (106 [48.2%] vs 84[49.1%]; difference, −0.9%; *P*= .85) or III/IV stage complications (20[9.1%)] vs 11[6.4]; difference, 2.7%; *P*=.34).

**Table 3:** Postoperative complications

Complication	Patients, no. (%) Surgery without IOC (***N***=220)	Surgery plus IOC (***N***=171)	***P*** value
Abdominal pain	84(38.2)	74(43.3)	.31
Hypoproteinemia	32(14.5)	20(11.7)	.41
Anastomosis or intra-abdominal bleeding	2(0.9)	1(0.6)	.71
Anastomosis leakage Abdominal cavity abscess	9(4.1) 7(3.2)	3(1.8) 2(1.2)	.18 .18
Wound problems	24(10.9)	18(10.5)	.90
Intestinal obstruction	4(1.8)	3(1.8)	.92
Cardiac disease	7(3.2)	8(4.7)	.45
Deep vein thrombosis	4(1.8)	3(1.8)	.96
Pulmonary disease	11(5.0)	9(5.3)	.90
Clavien-Dindo classification^a^			
I/II	106(48.2)	84(49.1)	.85
III/IV	20(9.1)	11(6.4)	.34
V	0	0	

Abbreviation: *IOC* Intraoperative intraperitoneal chemotherapy. ^a^The Clavien-Dindo classification scheme is explained in Dindo et al. [21]

To determine whether IOC was independent prognostic factor associated with CRC clinical outcomes, a univariate and multivariate analysis was performed using the Cox proportional hazard model (Table 4). The risk variables included age, gender, tumor location, tumor size, pathological *N* stage, differentiation, vascular invasion, ASA stage, TNM stage, IOC, and POC. These factors were generally considered to be associated with prognosis of CRC. In the univariate analysis, IOC (HR 0.53, 95%CI 0.32–0.86, *P*=.01), pathologic *N* stage (HR 2.12, 95%CI 1.24–3.61, *P*=.006), and TNM stage (HR 226, 95%CI 1.37–3.73, *P*=.002) were significantly associated with survival, while vascular invasion, gender, age, tumor size, tumor location, POC, and ASA stage were not. However, POC and vascular invasion were considered worthy of further study because of the *P* values of which were approximate to 0.05. In the final multivariate Cox regression model, IOC and POC independent of other factors was associated with a favor prognosis in CRC patients with stage II/III (HR 0.50, 95%CI 0.30–0.82, *P*=.006; HR 0.53, 95%CI 0.33–0.85, *P*=.009).

**Table 4:** Univariate and multivariate associations between covariates and the composite primary endpoint of recurrence or dead in CRC patients with stage II/III

Characteristic	Univariate analysis		Multivariate analysis	
	HR (95% CI)	***P*** value	HR (95% CI)	***P*** value
**IOC**				
Did not receive	1.0 (reference)		1.0(reference)	
Received	0.53(0.32–0.86)	.01	0.50(0.30–0.82)	.006
**Gender**				
Female	1.0(reference)		1.0(reference)	
Male	1.31(0.80–2.13)	.29	1.37(0.83–2.27)	.22
**Age**				
<50	1.0(reference)		1.0(reference)	
≥50	1.54(0.71–3.34)	.28	1.50(0.67–3.32)	.33
**Tumor location**				
Right colon	1.0(reference)		1.0(reference)	
Left colon	1.42(0.75–2.69)	.28	1.33(0.68–2.59)	.40
Rectal	1.11(0.63–1.95)	.71	0.92(0.51–1.66)	.78
**Tumor size**				
<5 cm	1.0(reference)		1.0(reference)	
≥5cm	1.01(0.64–1.58)	.97	1.13(0.70–1.81)	.62
**Pathologic** ***N*** **category**				
N0	1.0(reference)		1.0(reference)	
N1	2.12(1.24–3.61)	.006	2.23(1.23–4.05)	.008
N2	2.64(1.39–5.00)	.003	2.84(1.37–5.88)	.005
**TNM stage**				
II	1.0(reference)		1.0(reference)	
III	2.26(1.37–3.73)	.002	2.37(1.33–4.20)	.003
**Differentiation**				
Well	1.0(reference)		1.0(reference)	
Moderate	0.88(0.22–3.59)	.86	0.44(0.10–1.94)	.28
Poor	0.80(0.15–4.38)	.80	0.33(0.05–2.05)	.24
**Vascular invasion**				
No	1.0(reference)		1.0(reference)	
Yes	1.55(0.99–2.43)	.06	1.34(0.81–2.22)	.26
**ASA**				
II	1.0(reference)		1.0(reference)	
III/IV	1.29(0.83–2.03)	.26	1.54(0.96–2.49)	.07
**POC**				
Did not receive	1.0(reference)		1.0(reference)	
Received	0.66(0.42–1.04)	.07	0.53(0.33–0.85)	.009

Abbreviation: *IOC* Intraoperative intraperitoneal chemotherapy, *ASA* American Society of Anesthesiologists

**Fig. 2:**
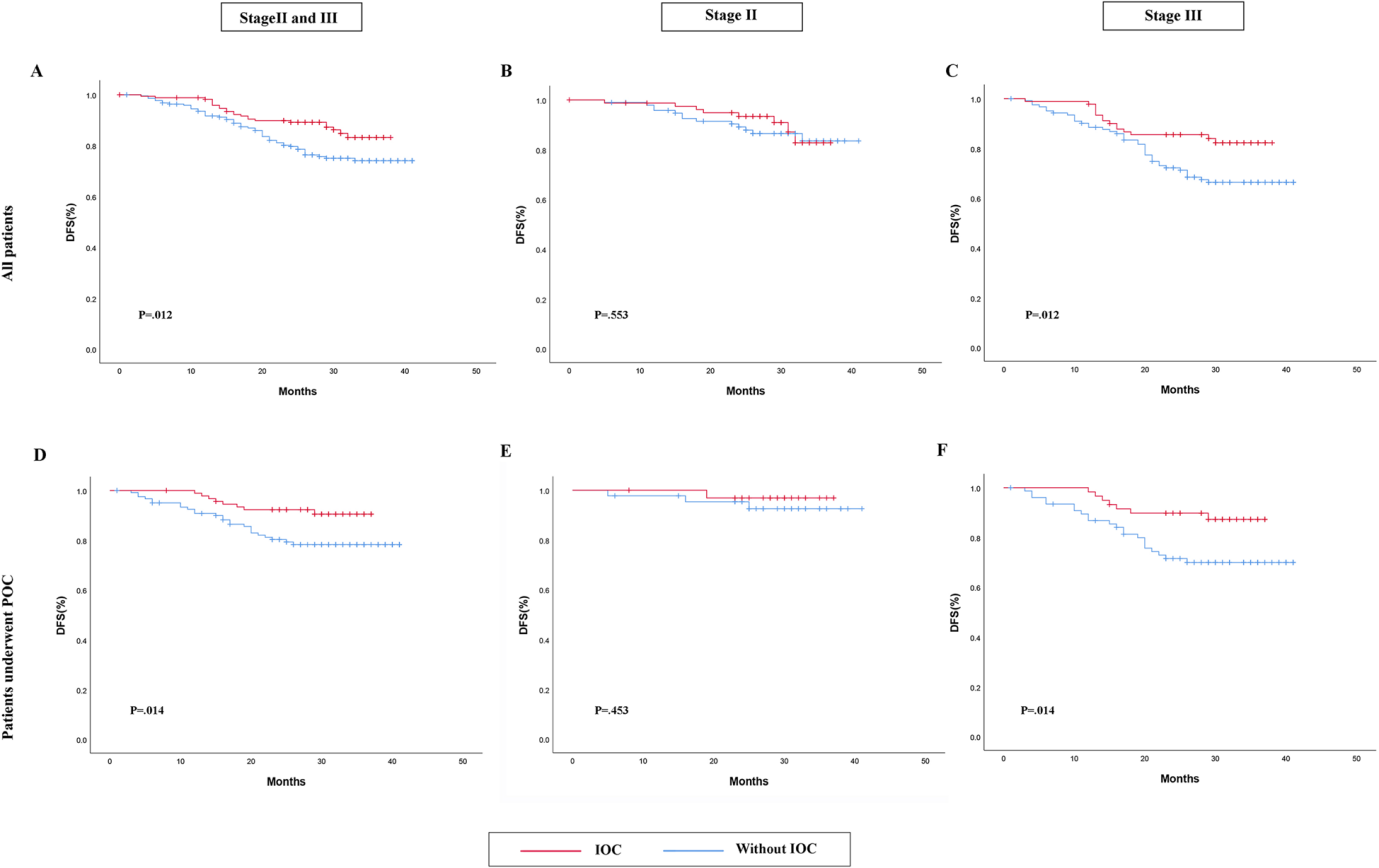
DFS in patients**. a–c** The association of DFS and IOC in all patients. **d**–**f** The association of DFS and IOC in patients who underwent POC. The red line is IOC. The blue line is without IOC. Abbreviation: IOC intraoperative intraperitoneal chemotherapy, DFS disease-free survival

**Fig. 3:**
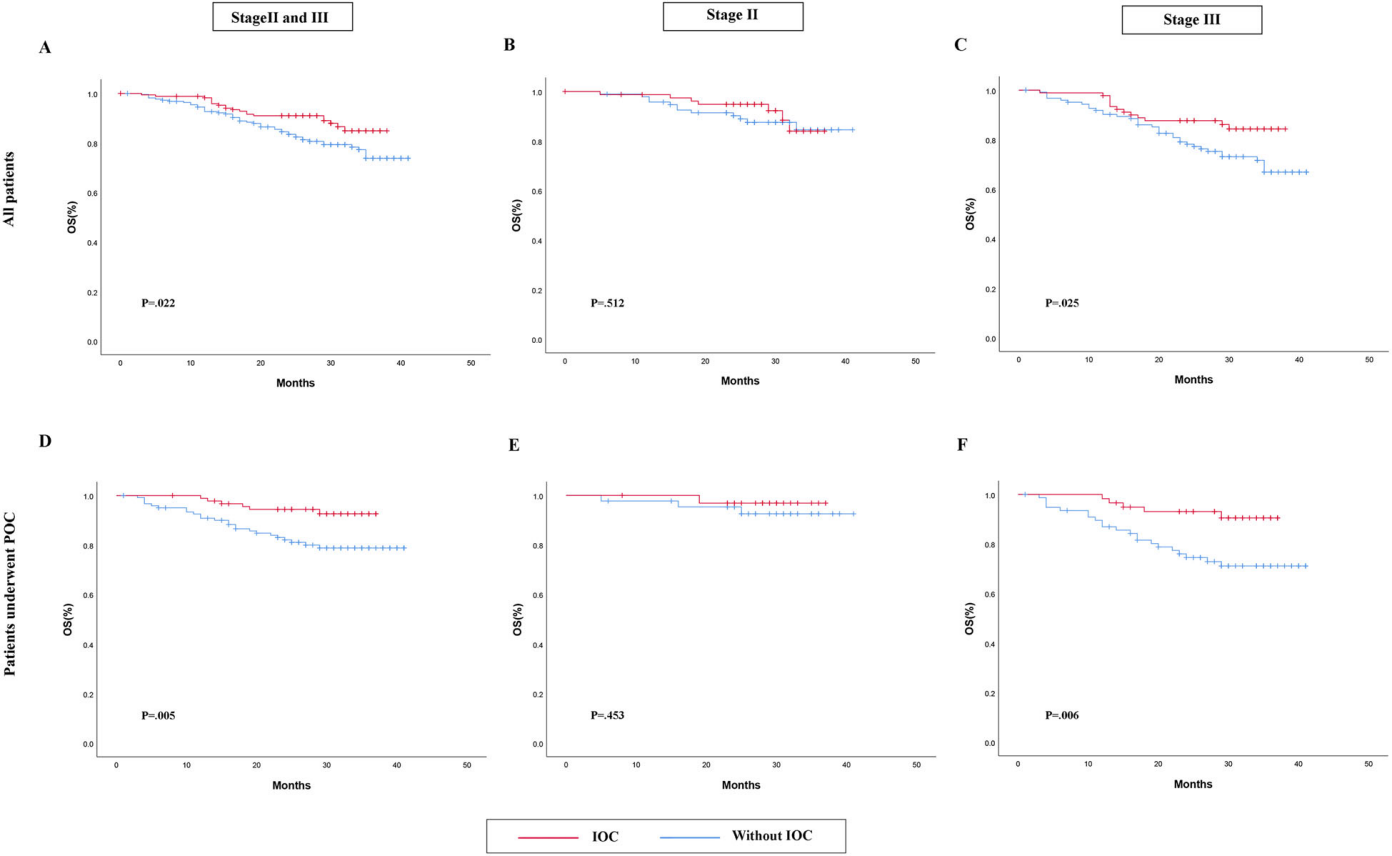
OS in patients. **a**–**c** The association of OS and IOC in all patients. **d**–**f** The association of OS and IOC in patients who underwent POC. The red line is IOC. The blue line is without IOC. Abbreviation: IOC intraoperative intraperitoneal chemotherapy, OS overall survival

In the present study, 75 patients had experienced a relapse or dead (surgery without IOC group, *N*=53; surgery plus IOC group, *N*=23). To further analyze the association of IOC and prognosis in patients with CRC, Kaplan-Meier analyses were performed. Considering the effect of prognosis with postoperative adjuvant chemotherapy (POC) in treating advanced colorectal carcinoma, POC was analyzed in the study. The POC regimens were mainly cisplatin combined with fluorouracil. The association between DFS and IOC was showed in Fig. 2. The survivorship analysis (Kaplan-Meier) showed a 83.2% DFS rate in IOC group and a 74% DFS rate in control group after 3 years (*P*=.012, Kaplan-Meier log-rank), and IOC was also associated with a favorable prognosis in patients underwent POC (*P*=.014, Kaplan-Meier log-rank). In patients with stage II colorectal carcinoma, DFS did not differ between two groups (*P*=.553, Kaplan-Meier log-rank), nor in patients accepted POC (*P*=.453, Kaplan-Meier log-rank). In stage III CRC patients, the estimated DFS rate for patients receiving IOC was 82.2% and patients without IOC was 66.4% after 3 years, which demonstrated that IOC was associated with a favorable prognosis in stage III patients (*P*=.012, Kaplan-Meier log-rank). Furthermore, the differences were still remained between the two groups when considering the influence about postoperative chemotherapy (*P*=.014, Kaplan-Meier log-rank). Moreover, the association of OS and IOC was shown in Fig. 3. Patients who underwent IOC perform a better prognosis than control group in stage II and III patients (*P*=.022, Kaplan-Meier log-rank), so did patients accept IOC combined POC (*P*=.005, Kaplan-Meier log-rank). In patients with stage II, IOC did not seem to make any sense to promote a better prognosis, neither they accept POC (*P*=.512, Kaplan-Meier log-rank) or not (*P*=.453, Kaplan-Meier log-rank). However, in patients with stage III, IOC can significantly improve patients’ overall survival whether they get treatment with POC (*P*=.025, Kaplan-Meier log-rank) (*P*=.006, Kaplan-Meier log-rank).

## Discussion

In recent years, the prognosis for colorectal cancer (CRC) performs more and more favorable with the development of diagnostic and treatment measures. However, the recurrence rate of patients who underwent curative resection for colorectal carcinoma was as high as 29.9% [22]. And peritoneal metastasis from colorectal cancer tended to perform an extremely poor prognosis. Remarkably, one survey revealed that the prognosis of the single-organ metastasis in the peritoneum group was even as poor as that of the multiple-organ metastases group [6].

Locally advanced colorectal cancer surgery, it should be noted, increases the risk of peritoneal metastasis because of the lymphatic opening caused by lymphatic clearance [8]. Therefore, if intraoperative intraperitoneal chemotherapy is put into effect, the viable malignancy cells resulting from destroyed lymphatic vessels may be eradicated and the prognosis would be better.

Here, we describe two main findings. First, when the lobaplatin concentration is 0.1g/L, there is no significant difference between the intraoperative intraperitoneal chemotherapy group and the control group in short-term complications rate. Second, whether the IOC group is superior to the control group depends on the stage of CRC. For stage II CRC patients, there were no distinct differences between the two groups in the terms of disease-free survival (DFS) and overall survival (OS); on the contrary, for stage III CRC patients, the IOC group has significant advantages both in disease-free survival (DFS) and overall survival (OS).

Several randomized prospective studies [12, 23] that evaluated the effect and safety of hyperthermic intraperitoneal chemotherapy (HIPC) as a therapy for peritoneal metastatic (PM) carcinoma have been published over recent decades. However, studies related to intraperitoneal chemotherapy during operation for CRC patients without PM are quite rare.

There are two literatures that evaluate the short-term efficacy of IOC in CRC patients. The study of the short-term effect analysis of intraoperative intraperitoneal perfusion chemotherapy with lobaplatin for colorectal cancer indicated that there is no distinction on short-term recovery between the study group and control group in patients with CRC, which was consistent with the result in the present study. However, Wang et al. indicated intraoperative intraperitoneal chemotherapy increases the incidence of anastomotic leakage after anterior resection of rectal tumors [24]. The lobaplatin concentration in Wang’s research was 0.12 g/L, which was higher than that in the present study, which may be the reason for the difference. Further studies are needed to verify whether IOC has an impact on postoperative anastomotic fistula for rectal malignant tumors. Moreover, one retrospective study evaluated the overall survival (OS) in CRC patients undergoing IOC. The research of intraoperative chemotherapy with a Novel Regimen Improved the Therapeutic Outcomes of Colorectal Cancer enrolled 551 CRC patients, of which 193 patients underwent IOC. There was no significant difference in complication rate and mortality between the two groups, but the IOC group presented a better prognosis in phase II and III CRC patients compared with the control group. Those results were basically consistent with the result in the present research. However, in the present study, we found there would be no significant difference between the two groups in terms of prognosis if the CRC patients were at stage II. The difference may result from the earlier T stage of patient in the present study. Of 182 stage II CRC patients, 174 (95.6%) patients were at T3 stage, and only 8 (4.4) patients were at T4 stage. Leung et al. presented that T4 stage identifies the majority of CRC patients who later develop PM [25]. Furthermore, in a prospective evaluation of the prognostic importance of peritoneal involvement in colonic cancer, Shepherd et al. enrolled 412 colorectal malignant tumor patients who underwent a primary resection and concluded that local peritoneal involvement is an independent predictive factor for intraperitoneal recurrence [26]. In addition, Jayne et al. found that 349 of 3019 patients with CRC have synchronous or metachronous PM, with 19% of the metachronous PM existing serosal invasion (stage T4) [5]. Therefore, intraoperative intraperitoneal chemotherapy should be recommended in patients with pT4 colorectal cancer. But further study was needed to demonstrate the effectiveness of IOC for T4 stage patients.

In the present study, lobaplatin was used for interoperative intraperitoneal chemotherapy. Lobaplatin, as a new generation platinum compound, has the same inhibitory effect on CRC cells as oxaliplatin [27]. In addition, lobaplatin is appropriate for intraoperative intraperitoneal chemotherapy due to its lighter inhibitory effects on the neurological system, hematological system, and gastrointestinal system. A preclinical model research [16] conduct that the survival rate of suspended CRC cells was only 16.3% when treated with 100mg/L lobaplatin for 6 h. Therefore, 0.1g/L lobaplatin intraperitoneal chemotherapy for 6 h during surgery was performed in order to obtain a strong efficacy on CRC patients with suspicious PM in the present study, while the dosage of 50 mg/person was far lower than the recommended dosage (50mg/m^2^), without any obvious toxic side reaction.

The present study had several limitations. First, this is a retrospective comparison, unobserved confounders remained. An RCT would be idealized. Second, a sample size of the retrospective study was still small because the duration of implementing intraperitoneal perfusion chemotherapy was less than 5 years in The Second Affiliated Hospital of Jilin University. But the study with a sample size of 391 patients was also acceptable. Third, our study involved only a Chinese population at a single center. Fours, there was no differences in the dosage of lobaplatin between CRC patients. Therefore, an individual chemotherapy regimen calculated by peritoneal area should be put forward through further study, though 50mg/L lobaplatin had been confirmed safe and effective. In addition, a prospective and multi-center study with a large sample size is required in the future.

## Conclusion

To our knowledge, only one study reported that IOC significantly improved the prognosis of colorectal cancer so far. In the present study, we found that surgery plus IOC generate a favorable prognosis for stage III CRC patients but not stage II without any side-effects when the dosage of lobaplatin was 0.1g/L. As a new, safe, and simple procedure, IOC therapy can be performed at most hospitals and does not require any special devices or techniques. Thus, IOC is a promising and exciting therapeutic strategy for patients with CRC.

## Data Availability

The datasets generated and analyzed during the current study available from the corresponding author on reasonable request.

## Abbreviations

IOC: Intraoperative intraperitoneal chemotherapy
POC: Postoperative adjuvant chemotherapy
CEA: Carcinoembryonic antigen
CRC: Colorectal cancer
DFS: Disease-free survival
CRS: Cytoreductive surgery
HIPEC: Hyperthermic intraperitoneal chemotherapy
OS: Overall survival
LOS: Length of stay
VAS: Visual analogue scale/score
SS: Saline solution
ASA: American society of anesthesiologists
PM: Peritoneal metastatic
PFCCs: Peritoneal-free cancer cells
